# RADx^SM^ Tech: A New Paradigm for MedTech Development Overview of This Special Section

**DOI:** 10.1109/OJEMB.2021.3070826

**Published:** 2021-06-01

**Authors:** Steven C. Schachter, John A. Parrish

**Affiliations:** ^1^ Co-PI of the Coordinating Center for POCTRNChief Academic Officer of CIMIT Boston MA 02114 USA; ^2^ Co-PI of the Coordinating Center for POCTRNChief Executive Officer of CIMIT Boston MA 02114 USA

This Special Section of the IEEE Open Journal of Engineering in Medicine and Biology focuses on the major interrelated components of Rapid Acceleration of Diagnostics (RADx^SM^) Tech, a National Institutes of Health (NIH)-funded program launched on April 29, 2020 to accelerate development, validation, and commercialization of innovative point-of-care and home- based tests, as well as improvements to clinical laboratory tests, that can directly detect SARS-CoV-2, the virus that causes COVID-19. RADx Tech was implemented and coordinated by the Consortia for Improving Medicine with Innovation & Technology (CIMIT) in its role as the Coordinating Center for the Point-of-Care Technologies Research Network (POCTRN) in conjunction with the NIH and the four POCTRN Centers [1].

Likened to a mini-Manhattan Project by Sen. Lamar Alexander [2], and as described throughout this Special Section, RADx Tech has proven to be unprecedented in many aspects, including its mission and vision, budget, accelerated timeframe, scale, extent of cross-government agency collaboration and information exchange, and blending of best business/academic/investment practices, all of which came together despite significant constraints imposed by the pandemic.

This Special Section provides operational details for the key components and processes of RADx Tech that have proven to be vital to its success: software platforms that enabled the program's infrastructure and processes (Collins *et al.*), the expert review panels (Tessier *et al.*), the unique facilitation provided to the funded applicants (Dempsey *et al.* and Robinson *et al.*), the POCTRN Cores that evaluated the technologies at the benchtop and in actual use (Nehl *et al.* and Gibson *et al.*), and the support for large-scale manufacturing and deployment of diagnostic tests (Walsh *et al.*). The final paper describes the impact of RADx Tech on future med-tech entrepreneurs and developers (DiMeo *et al.*).

On behalf of POCTRN and as Co-Principal Investigators for the POCTRN Coordinating Center, we are grateful to NIH leadership for their invaluable support and guidance, especially National Institute of Biomedical Imaging and Bioengineering director Bruce Tromberg and NIH director Francis Collins, and are privileged to present this collection of outstanding papers from a group of dedicated colleagues committed to serving our nation, which we humbly suggest represents a new paradigm for medical technology development and a validated model for the United States to use and adapt when challenged by another national healthcare emergency.


Steven C. Schachter, Guest Editor
Co-PI of the Coordinating Center for POCTRN
Chief Academic Officer of CIMIT
Boston, MA 02114 USA
sschacht@bidmc.harvard.edu

John A. Parrish
Co-PI of the Coordinating Center for POCTRN
Chief Executive Officer of CIMIT
Boston, MA 02114 USA

